# Effect of Advanced Glycation End Products on Human Thyroglobulin's Antigenicity as Identified by the Use of Sera from Patients with Hashimoto's Thyroiditis and Gestational Diabetes Mellitus

**DOI:** 10.1155/2015/849615

**Published:** 2015-07-02

**Authors:** A. Hatzioannou, I. Kanistras, E. Mantzou, E. Anastasiou, M. Peppa, V. Sarantopoulou, P. Lymberi, M. Alevizaki

**Affiliations:** ^1^Immunology Laboratory, Immunology Department, Hellenic Pasteur Institute, 127 Vasilissis Sofias Avenue, 11521 Athens, Greece; ^2^Endocrine Unit Athens University, Evgenideion Hospital, 20 Papadiamantopoulou Street, 11528 Athens, Greece; ^3^Endocrine Unit, Department of Medical Therapeutics, Alexandra Hospital, Athens University School of Medicine, 80 Vasilissis Sofias Avenue, 11528 Athens, Greece; ^4^Department of Geriatrics, Mt Sinai School of Medicine, 1468 Madison Avenue, New York, NY 10029, USA

## Abstract

Advanced glycation end products (AGEs) are formed on proteins after exposure to high concentrations of glucose and modify protein's immunogenicity. Herein, we investigated whether the modification of thyroglobulin (Tg) by AGEs influences its antigenicity and immunogenicity. Human Tg was incubated *in vitro* with increasing concentrations of D-glucose-6-phosphate in order to produce Tgs with different AGE content (AGE-Tg). Native Tg and AGE-Tgs were used in ELISA to assess the serum antibody reactivity of two patient groups, pregnant women with gestational diabetes (GDM), and patients with Hashimoto's thyroiditis (HT). We produced *in vitro* AGE-Tg with low and high AGE content, 13 and 49 AGE units/mg Tg, respectively. All HT patients' sera presented the same antibody reactivity profile against native Tg and AGE-Tgs, indicating that the modification of Tg by AGEs did not alter its antigenicity. Similarly, the GDM patients' sera did not discriminate among the two forms of Tg, native or artificially glycated, suggesting that the modification of Tg by AGEs might not alter its immunogenicity. The modification of Tg by AGEs has no obvious effect on neither its antigenicity nor, most likely, its immunogenicity. It seems that other Tg modifications might account for the production of aTgAbs in patients with GDM.

## 1. Introduction

Advanced glycation involves a chain of chemical reactions initiated by the nonenzymic, covalent binding of reducing sugars to protein amino groups (Schiff base and Amadori adducts). Additional reactions take place, leading to the formation of a heterogeneous family of sugar-amino acid adducts, collectively known as advanced glycation end products (AGEs) [1]. N-(carboxymethyl)lysine (CML) is a commonly formed AGE. Glycated hemoglobin (HbA1c) is one of the most studied glycated proteins, derived from the nonenzymic reaction between the valine and lysine amino groups of hemoglobin and glucose [2], and is commonly used in clinical practice as an indicator of glycemic control [3, 4]. Recent studies point towards a strong association between AGEs and HbA1c serum levels [5, 6].

The spontaneous modification of proteins by AGE formation occurring* in vivo *may increase their immunogenicity and lead to the induction of specific antibodies, such as antibodies against AGE-modified serum albumin, IgG, low-density lipoprotein (LDL), and factor VIII, as demonstrated in the sera from patients with diabetes or rheumatoid arthritis, as well as from healthy subjects [7–12]. The modification of proteins by AGEs has been suggested to be responsible for some of the complications encountered in diabetic patients; the immune complexes formed between AGE-LDL and anti-AGE-LDL antibodies may participate in the pathogenesis of atherosclerosis in diabetes mellitus [12]. We have recently observed in a preliminary study that in a group of women with gestational diabetes mellitus (GDM), a disorder characterized by mild hyperglycemia, those that presented anti-thyroglobulin antibodies (aTgAbs) also had significantly higher HbA1c levels.

Based on the above findings, we aimed to investigate the effect of AGEs on the antigenicity and immunogenicity of thyroglobulin (Tg), the main autoantigen of the thyroid gland, in order to understand the mechanisms underlying the production of aTgAbs in women with GDM. Here we describe the methodology used to prepare glycated thyroglobulin and the application of this preparation for the detection of specific antibodies in human sera.

## 2. Materials and Methods

### 2.1. Serum Samples

For the study of the correlation of the presence of aTgAbs with HbA1c levels we used 133 women with GDM at their 8th to 35th pregnancy week. For the study of the alteration of antigenicity and immunogenicity of Tg modified by glucose we used sera from three groups: (1) twenty pregnant women with GDM at their 8th to 35th pregnancy week. GDM was controlled either by diet or by insulin. The diagnosis of GDM was based on the ADA criteria 2000 [1]: glucose at fasting ≥ 95 mg/dL, 1 h ≥ 180 mg/dL, 2 h ≥ 155 mg/dL, and 3 h ≥ 140 mg/dL. Detection of two or more abnormal values led to the diagnosis of GDM. All of these women were euthyroid (mean free T4 14.32 ± 2.22 pmol/L, mean T3 1.25 ± 0.22 ng/mL, and TSH 1.6 ± 0.7 mU/L) but presented increased serum levels of aTgAbs [>70 International Units (IU)/mL]. Of the 20 women with GDM who were positive for aTgAbs, 18 also had positive anti-thyroid peroxidase antibodies (aTPOAbs). Fourteen of the women also reported positive family history for autoimmune disease. The majority of them were not aware of the presence of thyroid autoimmunity; (2) twenty female patients with Hashimoto's thyroiditis (HT) that had high serum aTgAbs titers and normal levels of HbA1c; and (3) twenty age-matched healthy females with normal aTgAbs and HbA1c serum levels. Free T4, T3, and TSH were determined by chemiluminescence immunometric assays with the DPC Immulite 2000 (Siemens, Gwynedd, UK) (Nichols Institute Diagnostics, San Juan Capistrano, California, USA). The reference range was for TSH 0.36–4 *μ*IU/mL, fT4 9–26 pmol/L, and T3 1.1–2.9 nmol/L. Pathological sera as well as sera from age-matched healthy donors, without any record of diabetes or thyroid disease in their first degree relatives were collected at the Endocrine Unit of the Department of Medical Therapeutics of Alexandra General Hospital. The protocol was approved by the Hospital's Ethics Committee. Informed consent was obtained by all women who participated in the study.

### 2.2. Assessment of aTgAbs, aTPOAbs, and HbA1c Serum Levels

Serum aTgAbs levels were assessed by Enzyme-Linked Immunosorbent Assay (ELISA) using a commercial kit (Boehringer Mannheim, Germany). The cut-off point of the assay was established at 70 IU aTgAbs/mL of serum. Serum aTPOAbs was determined by radioimmunoassay using the reagents Brahms DINOtest (Brahms diagnostic GmbH, Berlin, Germany). Reference ranges were aTPOAbs <60 IU/mL. Plasma glucose levels were determined by the glucose oxidase method (Integra400plus, Roche). HbA1c was determined using high pressure liquid chromatography (HPLC-HA8160 Menarini Arkay, Italy, method standardized to the DCCT assay, reference range for pregnancy from 4.3 to 5.5%). The inter- and intra-assay coefficients of variation (CV) for these parameters were all <5%.

### 2.3. Preparation of Tg from Human Thyroid Gland

Tg was extracted from the healthy portion of human thyroid glands dissected from patients operated for papillary thyroid cancer, without any abnormality in glucose metabolism (kindly provided by the surgeon of the Athens General Clinic N. Kakaviatos) according to a standard methodology [13]. Briefly, iced thyroid gland parts were homogenized in 0.15 M KCl (Merck, Germany) containing 1 mM PMSF (Merck) and 0.01% NaN_3_ (Merck). Tg was purified from thyroid extracts by precipitation with 1.52 M and 1.76 M ammonium sulphate (Merck) and gel filtration on Sephacryl S-300 (Pharmacia, UK) using phosphate buffer saline 10 mM pH 7.4 containing 0.15 M NaCl (Merck) (PBS). Tg purity was assessed by sodium dodecyl sulfate polyacrylamide gel electrophoresis (SDS-PAGE) under nonreducing conditions [14] and its concentration was determined spectrophotometrically at 280 nm (extinction coefficient *ε* for Tg = 1.0).

### 2.4. Preparation of AGE-Tg

For the preparation of AGE-Tg with low AGE content we used a previously described method for the production of AGE-modified bovine serum albumin (BSA) [15], slightly modified regarding glucose concentration and pH. In detail, Tg isolated from human thyroid glands (1.3 mg/mL, in PBS) was incubated with decreasing concentrations (0.11–0.004 M in PBS) of D-glucose 6-phosphate (Sigma Aldrich, USA) (with prior pH adjustment at 7.4 with NaOH) and EDTA (0.5 mM) (Merck) at 37°C, for seven weeks in the dark, under sterile conditions. The resulting pH was assessed and adjusted—if necessary—on a weekly basis. After incubation, low molecular weight reactants and glucose were removed by extensive dialysis (membrane's porosity 12000 Da) against PBS. Protein concentration was determined by Bradford assay (Bio-Rad, USA), by utilizing BSA for the standard curve. The effective AGE modification of Tg was evaluated qualitatively by SDS-PAGE under nonreducing conditions [14] and quantitatively by a competitive ELISA, as described below.

### 2.5. Quantitative Determination of AGE Content of Tg by ELISA

The AGE content of AGE-Tg was determined by competitive ELISA, as previously described [16], with minor modifications. Briefly, 96-well polystyrene microtiter plates (Nunc, Denmark) were coated with 100 *μ*L/well of 3 *μ*g/mL CML-modified BSA (CML-BSA) (Cosmo BIO, USA) in carbonate-bicarbonate buffer 0.1 M, pH 9.6 and incubated overnight at 4°C. Wells were washed with PBS and then blocked with 150 *μ*L of PBS containing 1% casein (Sigma) for 1 h at 37°C. Then plates were incubated with competitors ((a) standard curve: CML-BSA 1000 to 1.85 *μ*g/mL and (b) samples: AGE-Tg or control native Tg, diluted 1/2 and 1/5 and sera from healthy controls diluted 1/5) diluted in PBS containing 0.02% Tween 20 (PBS-T 0.02%) mixed with an equal volume of anti-CML-BSA mouse monoclonal antibody (1 *μ*g/mL) (provided by Dr. Vlassara) diluted in PBS-T 0.02% containing 2% goat serum. Plates were incubated for 2 h at 37°C, following an incubation (1 h at 37°C) with anti-mouse IgG (heavy and light chain specific) antibody conjugated to alkaline phosphatase (MP Biomedicals, USA) diluted (0.12 *μ*g/mL) in PBS-T 0.02% and 1% goat serum. At the end 100 *μ*L of enzymic substrate p-nitrophenyl phosphate (pNPP) (Sigma) was added to each well; after 2 h the optical density (OD) of the p-nitrophenolate product was measured at 405 nm using the V1.3 Expert Plus microplate reader (Asys, Austria). All incubations were followed by careful washing with PBS-T0.1%. Results were expressed as units AGE/mg of Tg. One AGE unit/mL is equal to the average *μ*g/mL of CML-BSA included in sera of healthy controls.

### 2.6. Comparison of Antibody Reactivity of Sera against Native Tg and AGE-Tg by ELISA

ELISA was performed as previously described [17, 18]. Polystyrene microtiter plates (Nunc) were divided in two sections: the first half was coated with 10 *μ*g/mL of native Tg in 100 *μ*L of carbonate-bicarbonate buffer 0.1 M, pH 9.6 and the other half with the same concentration of AGE-Tg in the same buffer and incubated for 1 h at 37°C and then overnight at 4°C. Wells were washed with PBS, followed by blocking with 5% bovine serum (BS) in PBS (PBS-BS) for 1 h at 37°C. Each coated antigen was incubated with the same serum samples, added in threefold dilutions (1/50 to 1/1350) in PBS-BS-T 0.1% for 2 h, followed by the addition of alkaline phosphatase-conjugated goat anti-human IgG (*γ*-chain specific) (250 ng/mL) (Sigma) in PBS-BS-T for 1 h at 37°C. The pNPP enzymic substrate was added and the product was measured as described above. All incubations were followed by careful washing with PBS-T0.1%. All sera were simultaneously tested against the two antigens and their titration curves were compared.

## 3. Results

### 3.1. Correlations of aTgAbs Presence and HbA1c Levels

In a group of 133 consecutive GDM women, we identified 14 who were positive for aTgAbs. We compared the HbA1c levels between the group of positive aTgAbs GDM women and the group of negative aTgAbs GDM women. Women that were positive for aTgAbs presented significant higher levels of HbA1c (mean = 5,0071%, Standard Deviation = 0,76) compared to women negative for aTgAbs (mean = 4,5347%, Standard Deviation = 0,69) (*p* value when equal variances are assumed = 0,019 and when equal variances are not assumed = 0,043).

### 3.2. Modification of Tg by AGE Formation

**Figure 1:**
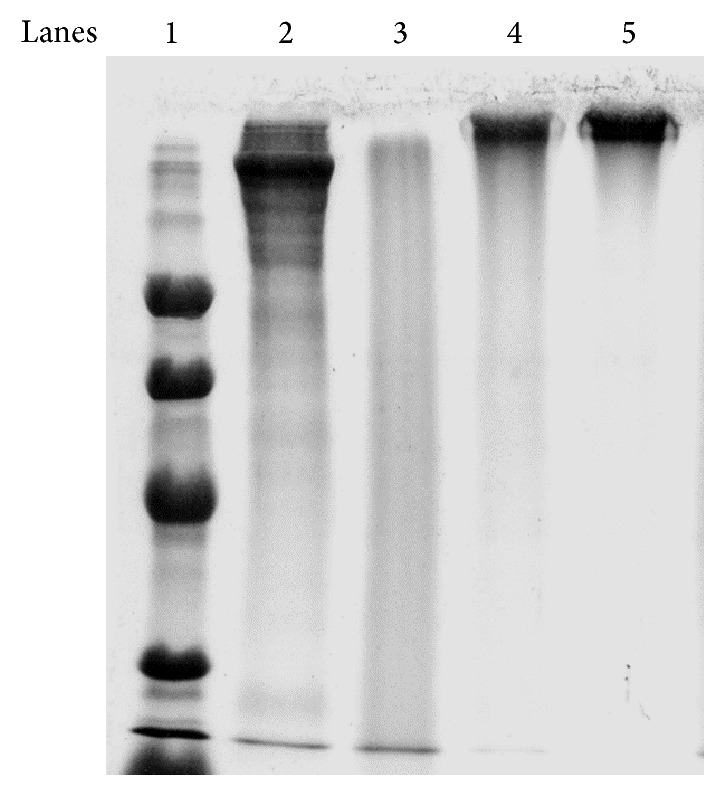
Analysis by SDS-PAGE of AGE-modified-Tg (in the presence of glucose, without pH adjustment at 7.4). Native Tg and AGE-Tg produced after incubation of Tg with three different concentrations of D-glucose-6-phosphate (no pH adjustment at 7.4) were analyzed by SDS-PAGE, samples of 10 *μ*g, in a 10% separating gel under denaturing, nonreducing conditions and then stained with Coomassie blue. Lane 1: molecular weight markers (94, 67, 43, and 30 KDa), lane 2: native Tg (330 KDa), lane 3: AGE-Tg produced with 0.03 M D-glucose-6-phosphate, lane 4: AGE-Tg produced with 0.05 M D-glucose-6-phosphate, and lane 5: AGE-Tg produced with 0.11 M D-glucose-6-phosphate.

**Figure 2:**
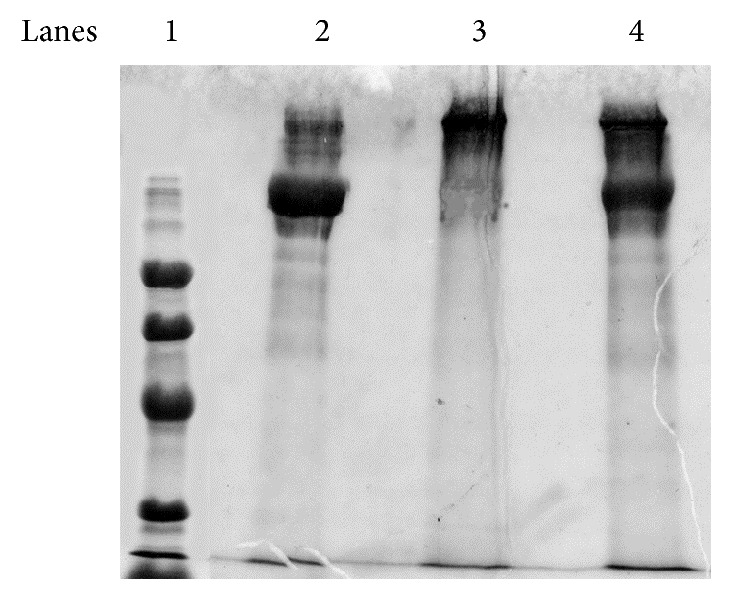
Analysis by SDS-PAGE of AGE-modified-Tg (in the presence of glucose, with pH adjustment at 7.4). SDS-PAGE analysis of native Tg and AGE-Tg produced after incubation of Tg with two different concentrations of D-glucose-6-phosphate (pH adjusted at 7.4). Samples (10 *μ*g) were analyzed in a 7.5% separating gel under denaturing, nonreducing conditions and then stained with Coomassie blue. Lane 1: molecular weight markers (94, 67, 43, and 30 KDa), lane 2: native Tg (330 KDa), lane 3: AGE-Tg produced with 0.03 M D-glucose-6-phosphate, and lane 4: AGE-Tg produced with 0.004 M D-glucose-6-phosphate.

We aimed to prepare a modified, low AGE content human Tg, via nonenzymic glycation, resembling the* in vivo* AGE content of proteins occurring in GDM [19]. In order to achieve Tg with low AGE content, we used D-glucose-6-phosphate concentrations (0.11, 0.05, and 0.03 M) lower than those applied in the glycation of BSA (0.5 M) [16]. When D-glucose-6-phosphate was added in the Tg solution, the pH instantly dropped to 1.0 and was adjusted to 7.4 with NaOH. Tg and D-glucose-6-phosphate were incubated for seven weeks at 37°C and the efficiency of the glycation was assessed by SDS-PAGE. The AGE content was measured by competitive ELISA. We found that the incubation of Tg with 0.03 M D-glucose-6-phosphate resulted in its degradation (Figure 1, lane 3), while Tg incubated with 0.05 M or 0.11 M D-glucose-6-phosphate remained intact (Figure 1, lanes 4 and 5). The AGE content of the AGE-Tg produced with 0.11 M D-glucose-6-phosphate was 49 units/mg Tg (high AGE-Tg) and 31 units/mg Tg with 0.05 M D-glucose-6-phosphate (Table 1). Apparently, the instant exposure of Tg to low pH combined with the low D-glucose-6-phosphate concentration resulted in degradation of the macromolecule. We further adjusted the pH of D-glucose-6-phosphate at 7.4 prior to incubation with Tg and tested its glycating efficiency at lower concentrations (0.03 M and 0.004 M). The AGE-Tg produced with the 0.03 M D-glucose-6-phosphate (low AGE-Tg) was more suitable for the study, since Tg presented the following characteristics: (1) it remained intact, (2) it was heavier (>330 KDa) than the native Tg (330 KDa) (Figure 2, lane 3 versus lane 2), and (3) its AGE content (13 units/mg Tg) was lower than that achieved with higher concentrations of glucose (49 units/mg Tg), but higher than that of native Tg (0.78 units/mg Tg) (Table 1). On the contrary, the AGE-Tg, produced with the 0.004 M D-glucose-6-phosphate, was partially glycated (two bands on an SDS-PAGE gel: 330 KDa and >330 KDa); therefore it was not suitable for the study, despite the fact that it was not being degraded (Figure 2, lane 4). Overall, the decrease in glucose concentration and the adjustment of its pH prior to incubation with Tg were the key points to ensure adequate Tg glycation with a low AGE content.

### 3.3. Effect of AGE Modification on the Antigenicity and Immunogenicity of Tg

In order to investigate the influence of the modification of Tg by AGE formation on its antigenicity and immunogenicity we tested the antibody reactivity of sera from women with HT and GDM, as well as from age-matched healthy controls, against high AGE-Tg (49 AGE units/mg Tg), low AGE-Tg (13 AGE units/mg Tg), and native Tg (0.78 AGE units/mg Tg). These antigens were immobilized under the same conditions on the same ELISA plates allowing us to compare serum reactivities against the different Tg preparations. Sera from women with HT were used to evaluate the antigenicity of AGE-Tg selected for their high content in aTgAbs (mean aTgAbs concentration ± SD = 1941 ± 983 IU/mL). The HT patients presented normal levels of HbA1c, which served as a marker for the levels of circulating glucose and those of nonenzymic glycation (4.6 ± 0.4%) (Table 2). Sera from women with GDM, seropositive for aTgAbs (exhibiting >70 IU/mL, mean aTgAbs concentration ± SD = 247.62 ± 297 IU/mL), were used to evaluate the immunogenicity of AGE-Tg. Their HbA1c levels (mean HbA1c ± SD = 5.4 ± 0.5%) were slightly higher compared to the cut-off point (5%) (Table 2). Control sera from healthy women exhibited normal levels of aTgAbs (<70 IU/mL) and HbA1c (4.6 ± 5.4%) (Table 2).

**Figure 3:**
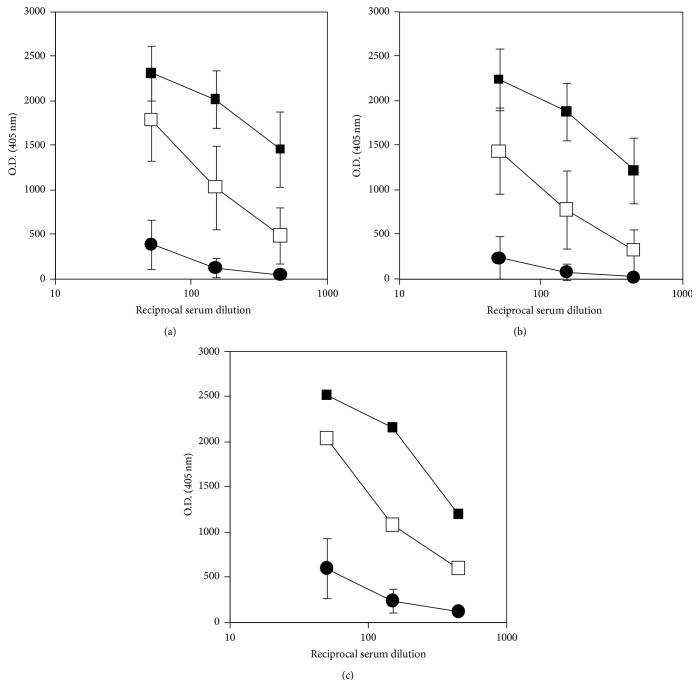
Titration curves of pathological (GDM and HT) and normal control sera against native Tg and low or high AGE-Tg. Threefold serial dilutions (1/50, 1/150, and 1/450) of 20 sera from pregnant women with GDM (open squares), 20 sera from women with HT (black squares), and 20 sera from healthy control women (black circles) were tested for antibody reactivity against native Tg (0,78 AGE units/mg Tg) (a), low AGE-Tg (13 AGE units/mg Tg) (b), and high AGE-Tg (49 AGE units/mg Tg) (c) by an in-house ELISA. Antibody reactivities were detected using alkaline phosphatase-conjugated anti-human IgG antibody and the adequate chromogenic substrate; mean OD values and SD (error bars) of sera from each group were calculated.

All twenty individual sera from GDM patients presented lower reactivity to all three Tg types, as compared to sera of HT patients, but higher reactivity than the sera from healthy individuals (Figure 3). It is therefore evident that the low extent of nonenzymic glycation of Tg cannot alter its antigenicity or offset the production of aTgAbs in GDM patients.

## 4. Discussion

It has been shown that accumulation of AGE on several proteins such as albumin, IgG, LDL, and factor VIII triggers specific immune responses and results in the production of anti-AGE antibodies or anti-AGE-modified protein antibodies [7–12]. The goal of the present study was the investigation of the effect of AGE modification of Tg on its antigenicity and immunogenicity.

Firstly, we indeed found that HbA1c levels were significantly higher in women with GDM that were positive for aTgAbs compared to women with GDM that were negative for aTgAbs. Next, we performed* in vitro* glycation of human Tg using a well-established protocol for BSA glycation [16]; we used, however, lower glucose concentrations, in order to obtain an AGE-modified Tg, similar to that which might in theory result from the* in vivo* glycation of Tg. Based on the fact that HbA1c serum levels of GDM patients are slightly higher compared to healthy individuals (5.4 ± 0.5% versus 4.6 ± 0.4%, resp.), we hypothesized that Tg from GDM patients will possess a low AGE content when modified* in vivo* by glucose. From our experiments it became evident that by lowering the glucose concentration, Tg was not protected against an instant exposure to the low pH of D-glucose-6-phosphate and was degraded. Thus, a crucial modification of the established protocol consisted in adjusting the pH of glucose to 7.4, which in turn resulted in an AGE-Tg with low content in AGE, that is, 13 units AGE/mg, comparable to that of HbA1c in diabetic patients, which ranges between 5.7 and 13.9 units AGE/mg protein [19].

In terms of Tg's antigenicity, the degree of glycation did not alter its recognition by HT patients' sera; high titer aTgAbs recognized native Tg, low AGE-Tg, and high AGE-Tg. This result indicates that the formation of AGEs on Tg does not mask any B-cell epitopes recognized by aTgAbs. A similar study on the effect of carbohydrate residues on the autoantibody binding sites of Tg also showed the inability of sugar residues to alter the autoantibody binding sites [20]. In order to investigate the effect of AGE modification on Tg's immunogenicity we compared the antibody reactivity of aTgAbs positive sera from women with GDM against native Tg and AGE-Tg. Based on our preliminary data, describing the correlation between HbA1c serum levels and the production of aTgAbs in pregnant women with GDM, we hypothesized that the modification of Tg by AGEs might lead to the production of aTgAbs in these patients. If this was the case, sera from women with GDM that exhibit higher levels of HbA1c than healthy individuals would only react with AGE-Tg and not with Tg. Our hypothesis was not verified on the present study; hence we cannot safely conclude on the role of AGEs on Tg's immunogenicity. Diabetic patients express glycated analogues of lens crystallins [21], insulin [22], LDL [12], collagen [23], myelin protein [24], histones [25], and apoprotein B [26], but the glycation levels of Tg have never been investigated. On the contrary, it has been shown that the oxidative stress caused by AGEs results in the production of immunogenic Tg fragments [27]. Another proposed mechanism explaining the association of TgAbs with AGEs is the general activation of the immune system that the latter induces. Indeed, the binding of AGEs on their receptor (RAGE) on T lymphocytes and on monocytes/macrophages stimulates the production of cytokines (interleukins, tumor necrosis factors, and interferons) and growth factors [28] while the AGE modification of several proteins induces the phenotypical and functional maturation of dendritic cells [29]. Moreover, it has been shown that increased glucose levels induce MHC class I expression on thyroid cells [30].

One limitation of our study is the fact that GDM may not represent the ideal population to study the effect of glycemia on the generation of aTgAbs. Future experiments should probably focus on models with higher degree of glycation. Despite the fact that these negative results do not have any clinical implications, it is important to note that we have shown that this methodology can be efficiently applied to test similar situations in other disease models. One would however need further experiments to show that* in vitro* glycation of Tg results in a similar Tg glycated* in vivo* in GDM patients.

## 5. Conclusions

Overall, we have shown that the modification of Tg by AGEs does not alter its antigenicity and therefore is unlikely to be the prime cause for the production of aTgAbs in women suffering from GDM. Perhaps, the use of diabetes and thyroiditis animal models would add to our knowledge on the effect of AGEs in the modification of the autoantigens.

## Figures and Tables

**Table 1:** Effect of modifications at the *in vitro *glycation method on Tg's AGE content.

	D-glucose pH adjustment^a^					
	No (pH = 1)				Yes (pH = 7.4)	
D-glucose concentration	0.11 M	0.05 M	0.03 M	0 M	0.03 M	0.004 M
Units AGE/mg Tg^b^	49	31	N.D.	0.78	13	N.D.

^a^D-glucose-6-phosphate solution pH was either adjusted or not to 7.4 prior to its mixing with Tg.

^b^Units AGE/mg Tg were determined by competitive ELISA.

**Table 2:** Average values ± SD of the parameters (age, HbA1c and aTgAbs, and aTPOAbs serum levels) in the group of patients with gestational diabetes mellitus (GDM), Hashimoto's thyroiditis (HT), and healthy controls (HC).

	GDM (*n* = 20)	HT (*n* = 20)	HC (*n* = 20)
Age (years)	34.2 ± 4.6	43.6 ± 15.5	29.4 ± 6.5
HbA1c%	5.4 ± 0.5	4.7 ± 0.4	4.6 ± 0.4
aTgAbs (IU/mL)	425.3 ± 455	1941.5 ± 983	<70
aTPOAbs (IU/mL)	236.66 ± 172.93	39311.11 ± 29359	<60

## Abbreviations

AGEs: Advanced glycation end products
Tg: Thyroglobulin
GDM: Gestational diabetes mellitus
aTgAbs: Anti-thyroglobulin antibodies
HT: Hashimoto's thyroiditis.
